# Suppressing glutamine metabolism in the pancreatic cancer microenvironment can enhance the anti-tumor effect of CD8 T cells and promote the efficacy of immunotherapy

**DOI:** 10.3389/fimmu.2025.1599252

**Published:** 2025-10-02

**Authors:** Jun Fan, Jianfei Chen, Rui Wang, Yisheng Peng, Sunde Tan, Xi Zhang, Hao Tang, Maoshan Chen, Bo Li, Xiaoli Yang

**Affiliations:** ^1^ Department of Breast and Thyroid Surgery, Suining Central Hospital, Suining, Sichuan, China; ^2^ Department of General Surgery (Hepatopancreatobiliary Surgery), The Affiliated Hospital of Southwest Medical University, Luzhou, China; ^3^ Academician (Expert) Workstation of Sichuan Province, Metabolic Hepatobiliary and Pancreatic Diseases Key Laboratory of Luzhou City, The Affiliated Hospital of Southwest Medical University, Luzhou, China; ^4^ Department of Thyroid and Breast Surgery, The First People’s Hospital of Zigong, Zigong, Sichuan, China; ^5^ State Key Laboratory of Molecular Vaccinology and Molecular Diagnostics, Center for Molecular Imaging and Translational Medicine, School of Public Health, Xiamen University, Xiamen, China

**Keywords:** pancreatic cancer, glutamine, CD8 T cells, immunotherapy, JHU083

## Abstract

**Objective:**

This study aims to investigate the relationship between tumor cell glutamine metabolism and CD8 T cells, with the goal of providing new insights to improve immunotherapy for pancreatic cancer.

**Methods:**

Using the The Cancer Genome Atlas – Pancreatic Adenocarcinoma (TCGA-PAAD) cohort, we computed gene expression scores related to glutamine metabolism and stratified patients into high- and low-score groups. Prognosis and differences in immune cell anti-tumor activity were compared between these groups. We further utilized single-cell RNA sequencing data to quantitatively assess the expression of glutamine metabolism-related pathways in tumor cells. Based on tumor-specific glutamine metabolism gene expression, patients were again classified into high- and low-score groups. The immune remodeling effects exerted by tumor cell glutamine metabolism on CD8 T cells were subsequently investigated. To examine the impact of perturbing glutamine metabolism within the tumor microenvironment on CD8 T cell phenotype and the efficacy of PD-1 inhibitors, we conducted *in vivo* animal experiments.

**Results:**

we analyzed the pancreatic cancer dataset in the cancer gene atlas database. We found that tumor glutamine metabolism was negatively correlated with patient prognosis and anti-tumor activity. Next, we defined two types of CD8 effector T cells in single-cell RNA sequencing data, namely, effector memory T cells (CD8-Tem) and terminally differentiated effector memory T cells (CD8-Temra). Under the pressure of high glutamine metabolism in tumor cells, the cytotoxicity of the CD8-Tem subset was reduced, and its immaturity score increased, while the exhaustion score of the CD8-Temra subset increased. Pseudotime analysis showed that CD8-Tn in the low-scoring group mainly developed into CD8-Tem subset, and its immune activation pathway was significantly upregulated. In addition, we found that the glutamine metabolism inhibitor JHU083 promoted the infiltration of CD4 and CD8 T cells and T lymphocyte differentiation, and increased the efficacy of PD-1 inhibitors. Glutamine inhibitors can inhibit the apoptosis of immune cells in the tumor microenvironment, while promoting CD8 T cells activation and cytotoxicity increase.

**Conclusion:**

Inhibition of glutamine metabolism within the pancreatic cancer microenvironment results in reduced apoptosis of immune cells, heightened activation and cytotoxicity of CD8 T cells, and a substantial enhancement in the therapeutic efficacy of immunotherapy.

## 1 Introduction

Pancreatic cancer is highly invasive, and patients have a poor prognosis (1–5). Currently, the efficacy of immunotherapy is unsatisfactory (6–16). T lymphocytes are the main immune cells infiltrating the tumor microenvironment of pancreatic cancer (17). CD8 T cells play a critical role in eliminating malignant cells and can provide long-term protective immunity (18–20). In tumor tissues, high abundance of CD8 effector T cells is positively correlated with the prognosis of pancreatic cancer patients (21, 22). However, CD8 T cells generally exhibit low infiltration and low cytotoxicity in pancreatic cancer (23–25). Existing studies have shown that the tumor microenvironment in which CD8 T cells reside is correlated with their developmental trajectory and determines their immune response. That is, the tumor microenvironment determines the anti-tumor ability of CD8 T cells (26). The classical theory has long held that tumor cells mainly obtain energy by taking up glucose in the immune microenvironment, and high glucose metabolism of tumor cells is a core factor that reshapes the metabolic microenvironment of tumors and prevents CD8 T cells from exerting their anti-tumor ability (27–29). Inhibition of glucose metabolism has long been regarded as an important strategy for treating tumors. However, effective inhibitors of glucose metabolism for tumor treatment have not been proven clinically so far. Recently, Professor Kimryn Rathmell’s research found that in the tumor immune microenvironment, tumor cells uptake more glutamine than glucose. At the same time, they found that immune cells in the tumor immune microenvironment are not lacking in glucose. In contrast, the amount of glutamine uptake by a single tumor cell is four times that of CD8 T cells (30). Therefore, it is possible that the high metabolism of glutamine in tumor cells in the tumor immune microenvironment leads to a change in the developmental trajectory of CD8 T cells, resulting in a decrease in their anti-tumor effect.

Rapidly proliferating cells, such as tumor cells, exhibit unique metabolic features to meet their high energy demands and increasing synthesis requirements for structural materials such as amino acids, nucleotides, and lipids, enabling sustained proliferation (31–34). Studies have also found increased expression levels of the glutamine transporter in various tumors (35), such as solute carrier family 1 member 5 (SLC1A5). The Myc oncogene can directly promote upregulation of SLC1A5 (36). These unique metabolic features increase the demand of tumor cells for glutamine (Gln) to promote synthetic metabolism. These findings suggest that tumor cells in the tumor microenvironment are dependent on glutamine. We may have overlooked the impact of tumor cell glutamine metabolism reshaping the tumor metabolic microenvironment on the phenotype of CD8 T cells.

Therefore, it is hypothesized that the anti-tumor effect of CD8T cell subpopulations may be diminished when pancreatic cancer remodels the tumor microenvironment through high glutamine metabolism. Disrupting such aberrant pancreatic cancer metabolic microenvironments may potentially enhance the infiltration and cytotoxicity of CD8 T cells, thereby increasing the efficacy of immune checkpoint inhibitors.It is worth noting that overcoming the immunosuppressive microenvironment often requires combination strategies. For example, accumulated evidence has shown that exercise can modulate a variety of cytokines, affect transcriptional pathways, and reprogram certain metabolic processes, ultimately promoting anti-tumor immunity and enhancing the efficacy of immune checkpoint inhibitors in cancer patients (37). Nonetheless, successfully targeting metabolic pathways or integrating adjunctive therapies remains challenging due to the highly complex and heterogeneous nature of the tumor microenvironment, which poses obstacles for designing selective and effective treatment strategies (38).

## 2 Methods

### 2.1 Source and data cleaning of pancreatic cancer tissue block sequencing data

The FPKM gene expression matrix of pancreatic cancer tissue block RNA sequencing data, as well as the corresponding clinical follow-up information, can be downloaded from the Cancer Genome Atlas database (index number TCGA-PAAD) (https://portal.gdc.cancer.gov/). All patients were diagnosed with pancreatic cancer through pathology. After excluding patients with missing clinical follow-up information, we obtained transcriptomic expression matrices of 176 patients and their corresponding clinical pathological parameters. By comparing with the genome annotation file GRCh38, we screened 18,965 protein coding genes and included them in the next analysis after removing duplicate probes.

### 2.2 Downstream analysis of pancreatic cancer tissue RNA sequencing data

According to the genes related to glutamine metabolism (ALDH18A1, GAPDH, GCLM, GLS, GOT1, MTHFS, OAT, SLC1A5, SLC38A1, SLC38A5, SLC7A5), we used the “ssGSEA” function in the “GSVA” package to calculate the expression scores of glutamine metabolism-related genes in tumor cells of each patient. Similarly, as cytotoxicity-related genes (GZMK, GZMH, GZMB, PRF1, IFNG, EOMES, NKG7), immune cell exhaustion-related genes (PDCD1, TIGIT, HAVCR2, LAG3, CTLA4), and immaturity-related genes (LEF1, SELL, TCF7, CCR7) are specifically expressed in immune cells, tissue block sequencing data can also be used to calculate the cytotoxicity scores of immune cell subgroups in each patient to evaluate the immune phenotype of immune cells in the immune microenvironment. After setting the median score of glutamine metabolism-related gene expression in patients’ pancreatic tissue as the grouping intercept value, 176 patients were divided into high and low score groups, and the relationship between the two groups and prognosis was explored.

### 2.3 The origin and data cleaning of single-cell RNA sequencing data

The single-cell RNA sequencing data used in this study were obtained from the Gene Expression Omnibus (GEO) with accession number GSE155698 (https://www.ncbi.nlm.nih.gov/geo/query/acc.cgi?acc=GSE155698) (39). Specifically, tumor tissues from 12 pancreatic cancer patients were selected for inclusion (4 patients were excluded due to fewer than 10 tumor cells). Cells with gene counts of 50 or more were included in downstream analysis if the same gene was expressed in at least 3 or more cells. Additionally, cells were excluded if their mitochondrial gene ratio was greater than 4%, ribosomal gene ratio was less than 2%, or hemoglobin gene ratio was greater than 10%. Finally, genes and cells meeting the aforementioned criteria were used for downstream analysis.

### 2.4 Clustering and biological annotation of single-cell RNA sequencing data

The software R (version 4.1.2) was utilized for the analysis of single-cell RNA sequencing data and tissue block sequencing data. The gene expression matrix of all cells was normalized using the built-in function “NormalizeData” from the Seurat package, with the scaling factor set to 10,000. The “vst” algorithm from the “FindVariableFeatures” function was employed to identify 3,000 highly variable genes. The expression matrix was then normalized using the “ScalData” function, with all genes used as reference genes. Principal component analysis was performed to identify statistically significant principal components (P-value < 0.05). To reduce data dimensionality, we used the t-distributed stochastic neighbor embedding algorithm with the top 15 principal components’ genes and performed clustering on all cells, with a resolution of 0.1. Based on molecular markers summarized in previous literature, we annotated the clustered cells as different biological subgroups, including neutrophils (ITGAM, ITGAX), epithelial cells (EPCAM, KRT18, KRT19), fibroblasts (TIMP1, FN1, ACTA2), mast cells (FCER1A, KIT), acinar cells (CTRB1, CELA3A, PLA2G1B), macrophages (CD68, CD163, LYZ), B cells (CD38, TNFRSF17), and NK and T cells (KLRB1, PRF1, CD2, CD3E, CD3D).

Identification of malignant epithelial cells: Since both malignant and normal epithelial cells express similar molecular markers, it is difficult to annotate the two subgroups based solely on differential gene expression. As malignant tumor cells originate from normal epithelial cells, the degree of malignancy often accompanies variations in chromosome structure and number. Therefore, in this study, we used the R package “infercnv” (https://github.com/broadinstitute/inferCNV) to calculate copy number variations (CNVs) in each of the 22 chromosomes of each cell based on its transcriptome, thereby defining malignant tumor cells and normal epithelial cells. The CNVs of each cell were sorted and classified by the position of the genes on the chromosome, and a moving average was applied to the relative expression values using a sliding window of 100 genes per chromosome. The reference cells were set as 1000 fibroblasts and 1000 T cells. Based on the obtained CNV matrix, an unsupervised clustering algorithm was used to divide all unidentified cells into multiple subgroups with varying copy numbers, with the subgroup with the lowest copy number and closest to the reference cell line defined as normal epithelial cells, and the rest defined as malignant tumor cells.

After extracting NK cell and T cell subpopulations, we used the ‘Harmony’ package to remove batch effects and reduce any unnecessary biological or technical factors. Next, the same standardization and dimension reduction procedures were applied to the T cell subpopulations. The functions ‘FindNeighbors’ and ‘FindClusters’ were used to identify individual cell subpopulations, with a resolution set at 0.8. The biological background of each subpopulation was annotated using known molecular markers. CD4 T cell subpopulations included CD4Tn (TCF7, SELL, IL7R, CCR7, LEF1, MAL), CD4Trg (FOXP3, PDCD1, CTLA4, TIGIT, BATF), CD4Tm (S100A4, S100A10, ANXA1, IL7R, KLF2), CD4Th17 (CCR6, IL2, DPP4, RORA, IFNGR1), and CD4Tfh (CXCL13, GNG4, CD200, IGFL2, TOX2). CD8 T cell subpopulations included CD8Tem (CD8 effector memory cells) (GZMK, GZMH, DUSP2, ITM2C, CD74, EOMES, CST7), CD8Trm (ZNF683, IL7R, ANXA1, CD55, GZMA, HOPX, CXCR6, ITGA1), CD8Temra (CD8 terminally differentiated effector memory cells) (GZMA, GZMH, GZMB, ZEB2, TBX21, NKG7, PLEK, KLRD1), CD8Tc17 (SLC4A10, CEBPD, NCR3, IFNGR1, RORA, LTK), and CD8Tn (CD8 naive T cells) (CCR7, LEF1, TCF7, SELL). The NK cell subpopulations included NK-FCGR3A (+) cells (NCAM1, CD160, FCGR3A) and NK-FCGR3A (-) cells (NCAM1, CD160). Some T cell subpopulations could not be mapped to known molecular markers after clustering (40) and were therefore not biologically annotated.

### 2.5 Patient grouping and pathway enrichment score calculation

Based on the glutamine metabolism-related genes (ALDH18A1, GAPDH, GCLM, GLS, GOT1, MTHFS, OAT, SLC1A5, SLC38A1, SLC38A5, SLC7A5), we calculated the expression scores of glutamine metabolism-related genes in tumor cells (GStumor) and CD8 T cells (GSimmune) using the “ssGSEA” algorithm in the “GSVA” package. After dividing the patients into high-score and low-score groups based on the median of GStumor scores from 12 patients, we compared the differences in the scores of cytotoxicity-related gene sets (GZMK, GZMH, GZMB, PRF1, IFNG, EOMES, NKG7), exhaustion-related gene sets (PDCD1, TIGIT, HAVCR2, LAG3, CTLA4), and naive-related gene sets (LEF1, SELL, TCF7, CCR7) between the different GStumor groups in CD8T subpopulations (CD8Tem and CD8-Temra).

### 2.6 Gene set enrichment analysis of tumor-infiltrating CD8 T cells

Gene Set Enrichment Analysis (GSEA) is a statistical method used to calculate the distribution trend of genes and determine their contribution to a specified phenotype, based on the comparison of sorted genes related to the phenotype and predefined gene sets. Compared to GO and KEGG enrichment analysis, GSEA can avoid the influence of subjective bias and retain more effective information, while also allowing for quantitative assessment of pathway activation. In this study, we downloaded multiple gene sets, including C2: CP: KEGG, C2: CP: REACTOME, and C5: GO (BP, MF, and CC) from the MsigDB website (https://www.gsea-msigdb.org/gsea/msigdb/). We selected the CD8Tem and CD8-Temra subsets, used the “FindAllMarkers” function to select differentially expressed genes that were upregulated and downregulated in the CD8Tem subset of patients in the low-score group, and calculated the fold change corresponding to these genes. Finally, we used the “GSEA” function in the “clusterProfiler” package to sort the gene sets according to fold change from high to low and perform enrichment analysis, obtaining enrichment scores for different pathways. The same method was used to process the CD8-Temra subset. We used the “AUCell_exploreThresholds” function to distinguish between high and low AUC values, which automatically defines the threshold for the bimodal distribution to determine the “activation” or “inactivation” status of cells in the relevant pathway gene set, respectively.

### 2.7 Under the influence of tumor cell glutamine metabolism, the developmental trajectory of tumor-infiltrating CD8 effector T cells

To investigate the differences of tumor-infiltrating CD8 effector T cells under different tumor cell glutamine metabolism pressures, we calculated the cytotoxic scores and cell proportions of these three CD8 T cell subsets (CD8-Tn, CD8-Tem, and CD8-Temra) and their changes between the two patient groups and performed pseudo-time gene dynamic analysis on the three CD8 T cell subsets (CD8-Tn, CD8-Tem, and CD8-Temra) using the “Monocle2” package in R. Monocle2 can use unsupervised machine learning and reverse graph embedding algorithms based on single-cell transcriptome expression matrices to place cells on different branches of the developmental trajectory to simulate the biological process of the cell population, forming a “one-root-two-branches” cell development tree diagram, in which cells on the same branch have the same gene expression features and differentiation status. This pseudo-time analysis can infer the differentiation trajectory of cells or the evolution process of cell subtypes during development, and identify key genes and pathway changes that affect branch formation. We extracted three objects, including gene expression matrix, gene information, and cell phenotype information, and constructed them into a “CellDataSet” object. The “estimateSizeFactors” function can standardize the transcriptome expression matrix. Using the “FindAllMarkers” function, we screened for upregulated genes in CD8-Tef (CD8-Tem and CD8-Temra) under these two metabolic modes, and then used the “DDRTree” algorithm to project all cells onto a two-dimensional plane and arrange them in order of branching.

### 2.8 Establishment, grouping, and drug intervention of a mouse model

A cell suspension of 0.1 ml at a concentration of 1×10^6/ml Panc02 tumor cells (i.e., 1×10^5 cells per mouse) was inoculated into the right groin area of each mouse. On day 6 post-inoculation, the length and width of the subcutaneous tumors in the mice were observed and recorded, and the volume of the subcutaneous tumors was calculated using the formula: V (mm^3) = length (mm) × width (mm) × width (mm) × π/6. Twenty mice with subcutaneous tumors of similar volumes were selected and randomly divided into four groups (five mice per group):

1. Control group (VEH): orally administered with 100 μL of 0.9% saline solution per day and intraperitoneally injected with 100 μL of 0.9% saline solution once every three days for 20 consecutive days.
2. Glutamine metabolism inhibitor group (JHU083): orally administered with 100 μL of JHU083 solution in saline (1 mg/kg/d) per day and intraperitoneally injected with 100 μL of 0.9% saline solution once every three days for 20 consecutive days.
3. Immune checkpoint inhibitor group (Anti-PD-1): intraperitoneally injected with 100 μL of PD-1 monoclonal antibody solution in saline (1 mg/kg/d) once every three days and orally administered with 100 μL of 0.9% saline solution per day for 20 consecutive days.
4. Combination of glutamine metabolism inhibitor and immune checkpoint inhibitor group (JHU083+Anti-PD-1): orally administered with 100 μL of JHU083 solution in saline (1 mg/kg/d) per day and intraperitoneally injected with 100 μL of PD-1 monoclonal antibody solution in saline (1 mg/kg/d) once every three days for 20 consecutive days.

The length and width of the tumors were measured every two days, and the volume of the subcutaneous tumors was calculated accordingly. All mice were euthanized after being fed for 27 days, and subcutaneous tumor samples were harvested immediately after euthanasia. To ensure humane euthanasia, mice were placed in a CO_2_chamber with a flow rate set at 30% of the chamber volume per minute, following approved welfare guidelines. The CO_2_concentration was gradually increased to induce unconsciousness, followed by respiratory and cardiac arrest.

### 2.9 Quantitative real-time polymerase chain reaction

Approximately 50 mg tumor tissue was grind and crushed, add an appropriate amount of Trizol lysis solution to it and lyse it thoroughly on ice. The lysate was then transferred to an enzyme-free EP tube and centrifuged at 4°C, 12,000 rpm/min for 10 min; the supernatant obtained by centrifugation was then transferred to another EP tube, chloroform was added, the supernatant and chloroform were mixed and left to stand for 15 min, next centrifuged at 4°C, 8,000 rpm/min for 15 min. Wash with 75% ethanol solution, centrifuge for 15 min at 4°C at 12000 rpm/min, add 20 μl DEPC water to the precipitate, wait for the precipitate to dissolve, measure the mRNA concentration. mRNA was collected and reverse transcribed into cDNA, which were amplified in triplicate using SYBR Green PCR Master Mix (Guangzhou RiboBio Co), 10 pmol of primer (**Supplementary Table S1**), and 20 ng of cDNA per reaction with the StepOnePlus (Roche LightCycler 96). Quantitation was performed using the ΔΔCt method.

### 2.10 Immunohistochemistry

All pathological diagnoses were made independently by 2 senior physicians in the Department of Pathology, and controversial diagnoses were assessed by a third physician and then decided by joint consultation. The specific steps of staining were as follows.

1. Dewaxing and hydration: The slices were placed in the oven at a temperature of 60°C for 90 min, then placed in xylene for 30 min for dewaxing, then the slices were immersed in ethanol (anhydrous ethanol, 95% ethanol, 75% ethanol) in a gradient from high to low concentration for 5 min, and finally rinsed repeatedly with double-distilled water for 5 min.
2. Antigen repair and peroxidase removal: The treated tissue sections were placed in a repair cassette with 200 ml of ethylene glycol tetraacetic acid (EDTA) solution, then placed in an autoclave with double-distilled water, first heated to vapour, then allowed to cool, and then rinsed with double-distilled water. The sections were then placed in 3% hydrogen peroxide solution (H2O2) for 10 min incubation protected from light, allowed to cool and then soaked 3 times with double distilled water for 5 min each and rinsed with PBS for 5 min.
3. Addition of antibody, colour development, re-staining and blocking: sections were added dropwise with antibody (KI67, CD3 and CD8) diluted at 1:200 and refrigerated overnight at 4°C. The next day the sections were washed three times with PBS for 5 min each time. Second day, the sections were washed three times with PBS for 5 min each time, shaken dry, incubated with secondary antibody for 30 min, and washed three times with PBS for 5 min each time. The reaction was terminated by adding a drop of DAB staining solution to the sections and observing a positive reaction under the microscope. After washing, the sections were fractionated with ethanol hydrochloride solution, then washed, dehydrated, sealed and labelled.
4. After the above steps were completed, the pathological sections were observed under an inverted fluorescent microscope. The expression levels of KI67, CD3 and CD8 proteins were measured with Image J software.

### 2.11 Flow cytometry

Immune cell populations were identified via flow cytometry from respective dissociated whole tumor cell suspensions.

(1) After mechanically cutting the tumor tissue, it was filtered with 300 mesh filter cloth, centrifuged with 300 g for 5 min, and the cell concentration was adjusted to 10*6/mL with PBS. (2) 1 µg antibody (CD8a, CD3, cd49b, CD45, CD4, LIVE/DEAD) were add into 100 µL cell suspension in the sterile EP tube. Dye at 4 °C for 30 min without light after mixing. (3) Adding 1000 μL PBS to wash the mixture, the supernatant was removed after centrifuging with 300g for 5min. (4) Cells were resuspended by 400 μL PBS and then detected by ZE5 flow cytometry, flow cytometry data were analyzed using FlowJo software.

### 2.12 Immunocyte apoptosis detection

(1) Take 100 μL of the immunocyte suspension separated from “Flow cytometry (1)” and centrifuge at 300g for 5 minutes. Discard the supernatant and resuspend the cells in 100 μL of binding buffer. (2) Add 5 μL of Annexin V-FITC staining fluorescent dye and incubate for 10 minutes at room temperature in the dark. (3) Add 10 μL of PI staining dye and incubate for 5 minutes at room temperature in the dark. Add 400 μL of PBS and resuspend the cells. Immediately detect the cells using a flow cytometer. (4) Analyze the data using FlowJo software and a ZE5 flow cytometer.

### 2.13 Statistical analysis

The statistical analysis of the experimental data was performed using R software (version 4.1.2) in accordance with the conventions of medical academic papers. In this study, Kaplan-Meier survival analysis was performed to compare overall survival (OS) among different groups. In this study, overall survival (OS) was defined as the time interval from the date of diagnosis (the starting point) to the date of death from any cause (the endpoint). For patients who were still alive or lost to follow-up by the time of analysis cutoff, their OS time was censored at the date of the last known follow-up. All OS and follow-up data were obtained from clinical follow-up records within the TCGA database. If the data were normally distributed, t-test was used for comparison. If not, Wilcoxon rank sum test was used instead. P value less than 0.05 was considered statistically significant. 0.05 ≤ P value < 0.10 as indicative of borderline significance. Wilcoxon and t-tests were adjusted for using the Benjamini–Hochberg method via the p.adjust function in R.

## 3 Result

### 3.1 The scoring of genes related to glutamine metabolism in tumor tissue is negatively correlated with patient prognosis and anti-tumor immune presentation

**Figure 1:**
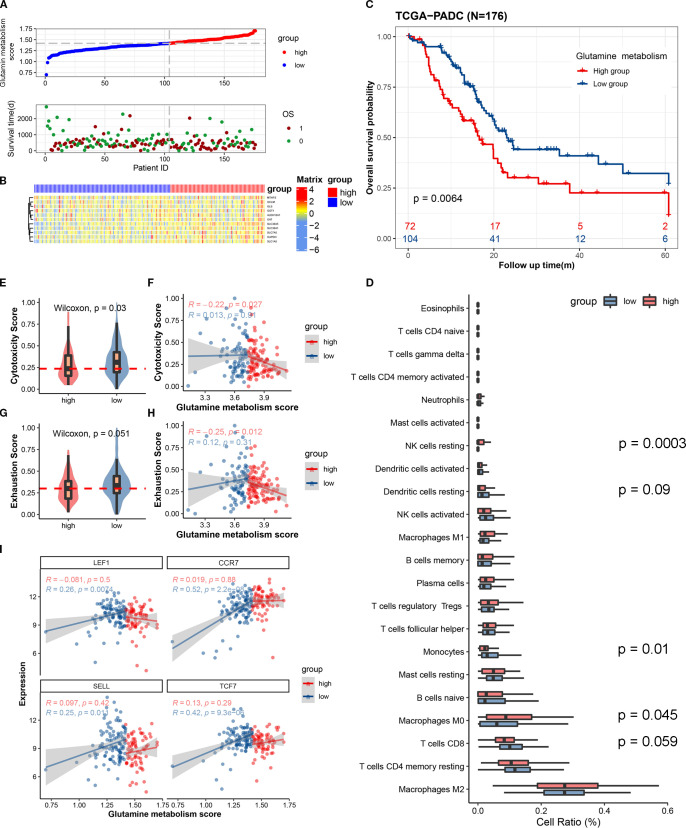
The expression scores of glutamine metabolism-related genes in the tumor microenvironment of pancreatic cancer are closely correlated with the anti-tumor activity of immune cells and the prognosis of patients. **(A)** The upper curve shows the distribution of glutamine metabolism-related gene expression scores in tumor cells of all pancreatic cancer patients. The lower dot plot shows the survival status and survival time of all patients sorted by tumor glutamine metabolism-related gene expression scores from low to high. **(B)** The heatmap shows the expression levels of glutamine metabolism-related genes between two groups. Blue represents low metabolism group, and red represents high metabolism group. **(C)** This Kaplan-Meier curve shows the difference in overall survival rate between different groups. **(D)** The box plot shows the degree of infiltration of 22 immune cells in the tumor microenvironment. (**E**, **G**) Differences in cell toxicity score and exhaustion score of CD8T cells between the two groups. (**F**, **H**) Fitting curves of glutamine metabolism score of tumors in the two groups with cell toxicity score and exhaustion score. The correlation coefficient and significance test were calculated and marked in the upper left corner. **(I)** Fitting curves of glutamine metabolism score of tumor cells in the two groups with expression levels of immature-related genes. The correlation coefficient and significance test were calculated and marked in the upper left corner.

To distinguish between high and low metabolism of glutamine and to clarify the relationship between glutamine metabolism levels and prognosis of pancreatic cancer patients, we scored the expression of glutamine metabolism-related genes (ALDH18A1, GAPDH, GCLM, GLS, GOT1, MTHFS, OAT, SLC1A5, SLC38A1, SLC38A5, SLC7A5) in tumor cells of 176 patients in TCGA cohort, and divided them into high and low scoring groups based on the median value (**Figure 1A**). Hierarchical clustering results also demonstrated the expression differences of glutamine metabolism-related genes between these two patient groups, indicating that unsupervised clustering algorithms can significantly separate these 176 pancreatic cancer patients based on glutamine metabolism-related genes (**Figure 1B**). Kaplan-Meier curves showed that patients in the high scoring group had a worse prognosis. In the high scoring group, the overall survival rates at 1, 3, and 5 years were 63.1%, 27.1%, and 22.6%, respectively. However, in the low scoring group, the overall survival rates at 1, 3, and 5 years were 82.1%, 41.0%, and 27%, respectively, suggesting a negative correlation between glutamine metabolism and pancreatic cancer prognosis (**Figure 1C**). The baseline characteristics of the patients are detailed in **Supplementary Table S3**.

In order to investigate whether the metabolism of glutamine in tumor cells affects the anti-tumor activity of immune cells in pancreatic cancer, we used single-sample gene set enrichment analysis (ssCSEA) to study the relationship between glutamine metabolism in tumor cells and immune infiltration, cytotoxic gene set (GZMK, GZMH, GZMB, PRF1, IFNG, EOMES, NKG7), immune exhaustion gene set (PDCD1, TIGIT, HAVCR2, LAG3, CTLA4), and immature-related gene set (LEF1, SELL, TCF7, CCR7) in the pancreatic cancer microenvironment. Immune infiltration analysis revealed a trend toward higher CD8+ T cell infiltration in the tumor immune microenvironment of the low glutamine score group compared to the high glutamine score group (P = 0.059) (**Figure 1D**). In the low score group, the immune cell cytotoxicity-related gene score was significantly higher than that in the high score group (**Figure 1E**). By plotting the glutamine score and immune cell cytotoxicity score of the two groups, we found that there was a negative correlation between tumor cell glutamine metabolism and immune cell cytotoxicity in the high score group (**Figure 1F**). In terms of immune exhaustion score, we found that the low score group was significantly higher than the high score group (**Figure 1G**), which indicates a negative correlation between tumor cell glutamine metabolism and immune cell exhaustion. To determine whether the low immune cell exhaustion score in the high glutamine score group is related to the level of immature immune cells, we plotted the glutamine score and immature-related gene set (LEF1, SELL, TCF7, CCR7) of the two groups, and found that as the glutamine score increased in tumors with high glutamine metabolism, the expression of immature immune cell genes increased significantly (**Figure 1I**). This may suggest that the decrease in immune cell cytotoxicity in the high glutamine metabolism group of tumor cells is related to the level of immature immune cells. The results of this study indicate that as the glutamine metabolism in tumors increases, immune cells become more immature, while the scores of immune cell cytotoxicity and exhaustion decrease.

### 3.2 Identification of subpopulations and patient stratification using single-cell transcriptome sequencing data

**Figure 2:**
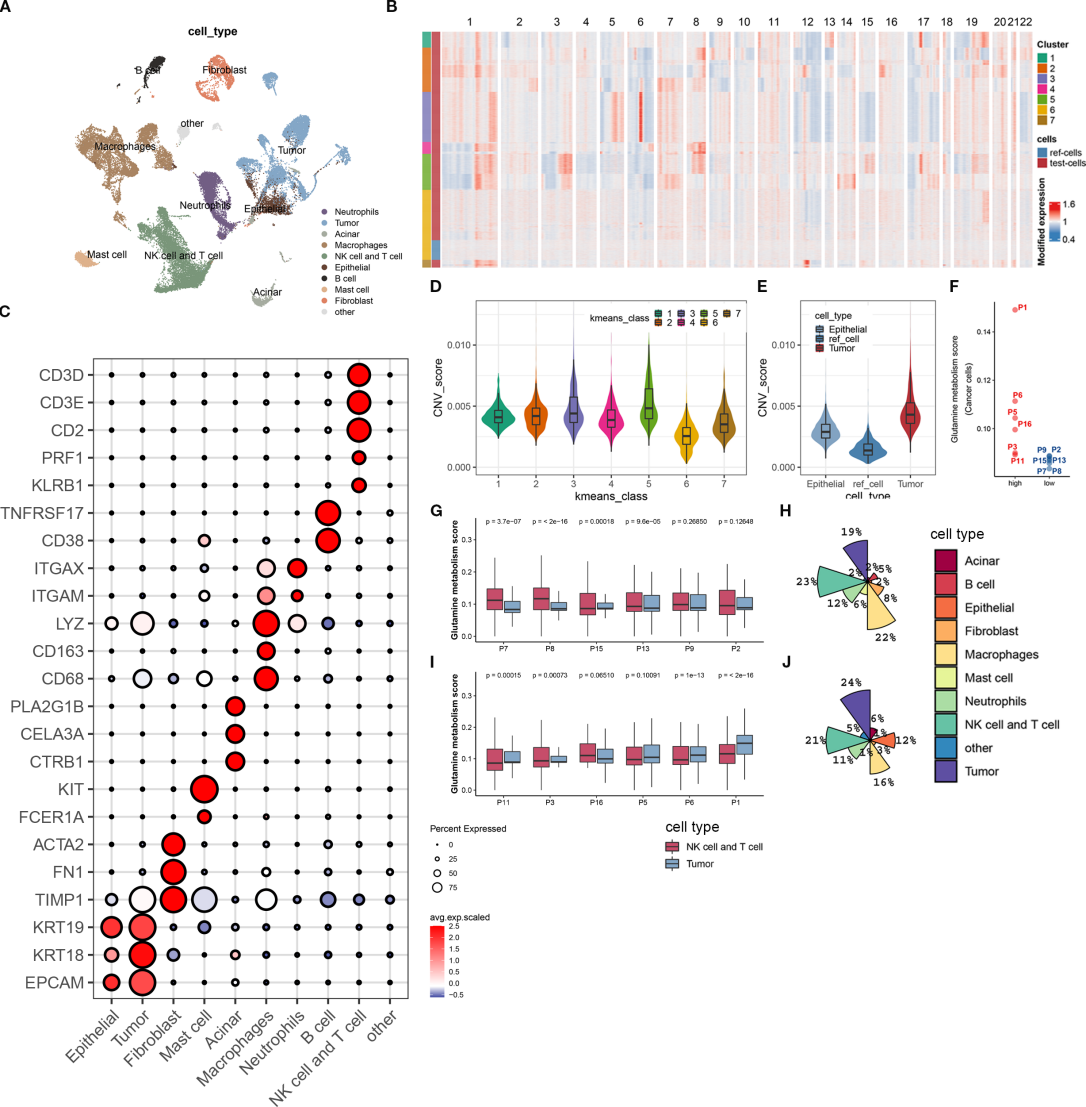
Biological annotation of single-cell sequencing data. **(A)** The t-SNE plot displays all the cellular subpopulations present in the tumor microenvironment of pancreatic cancer patients. Different colors represent different cell types. The cellular subpopulations are annotated as shown in the figure. **(B)** The heatmap shows copy number variations of all genes on 22 chromosomes in normal epithelial cells and malignant epithelial cells. All cells are classified into 7 groups using unsupervised clustering algorithm. The group with the lowest copy number variation is identified as normal epithelial cells, while the rest are malignant epithelial cells. **(C)** The bubble plot shows the expression levels of molecular marker genes and cell proportions in each cellular subpopulation. The color of the dot represents the average expression level of the gene, with red indicating high expression and blue indicating low expression; the size of the dot represents the cell proportion. **(D)** This boxplot shows the difference in copy number variation scores between the 7 epithelial cell subpopulations. **(E)** We demonstrate the difference in copy number variation scores between the reference cell line, normal epithelial cells, and malignant epithelial cells (tumor cells) after biological annotation. **(F)** This scatterplot divides 12 pancreatic cancer patients into high- and low-metabolism groups based on the expression score of glutamine metabolism-related genes in tumor cells. **(G, H)** The boxplot **(G)** displays the expression score of glutamine metabolism-related genes in tumor cells, T cells, and NK cells of 6 low-metabolism group patients, while the pie chart **(H)** shows the composition ratio of all cell subpopulations in this group. **(I, J)** The boxplot **(I)** displays the expression score of glutamine metabolism-related genes in tumor cells, T cells, and NK cells of 6 high-metabolism group patients, while the pie chart **(J)** shows the composition ratio of all cell subpopulations in this group.

To obtain a comprehensive single-cell gene expression atlas of the pancreatic cancer immune microenvironment, we used the program package ‘Seurat’ to identify differential genes and cell types in 12 pancreatic cancer patients. A total of 39,719 qualified cells were annotated as nine major cell subtypes and one undefined cell subtype (**Figure 2A**). The major cell subtypes include neutrophils (ITGAM, ITGAX), epithelial cells (EPCAM, KRT18, KRT19), fibroblasts (TIMP1, FN1, ACTA2), mast cells (FCER1A, KIT), acinar cells (CTRB1, CELA3A, PLA2G1B), macrophages (CD68, CD163, LYZ), B cells (CD38, TNFRSF17), and NK/T cells (KLRB1, PRF1, CD2, CD3E, CD3D) (**Figure 2C**). Tumor cells were identified using the INFERCNV algorithm (https://github.com/broadinstitute/inferCNV). As no known molecular markers were mapped to the undefined cell subtype, it was not included in this study.

In order to investigate the impact of tumor cell glutamine metabolism on immune subpopulations in the immune microenvironment of pancreatic cancer, we divided 12 patients into a high-scoring group (P01, P6, P5, P16, P3, P11) and a low-scoring group (P8, P2, P13, P15, P7, P8) based on the expression scores of glutamine metabolism-related genes in tumor cell subpopulations (**Figure 2F**). At the same time, we found that in the low-scoring group of tumor cell glutamine metabolism, the glutamine scores of T cells and NK cells were mostly higher than those of tumor cell glutamine scores. In contrast, the opposite was true in the high-scoring group of tumor cell glutamine metabolism (**Figures 2G, I**). Interestingly, compared with the low-scoring group, the high-scoring group had more tumor cells (24% vs 19%) and fewer T cells and NK cells (21% vs 23%) (**Figures 2H, J**). This suggests that in the immune microenvironment of pancreatic cancer, high tumor cell glutamine metabolism will be accompanied by low metabolism of immune cells and low infiltration of T cells and NK cells.

### 3.3 In the immune microenvironment of pancreatic cancer, the glutamine metabolism of tumor cells can affect the anti-tumor activity of CD8 T cells

**Figure 3:**
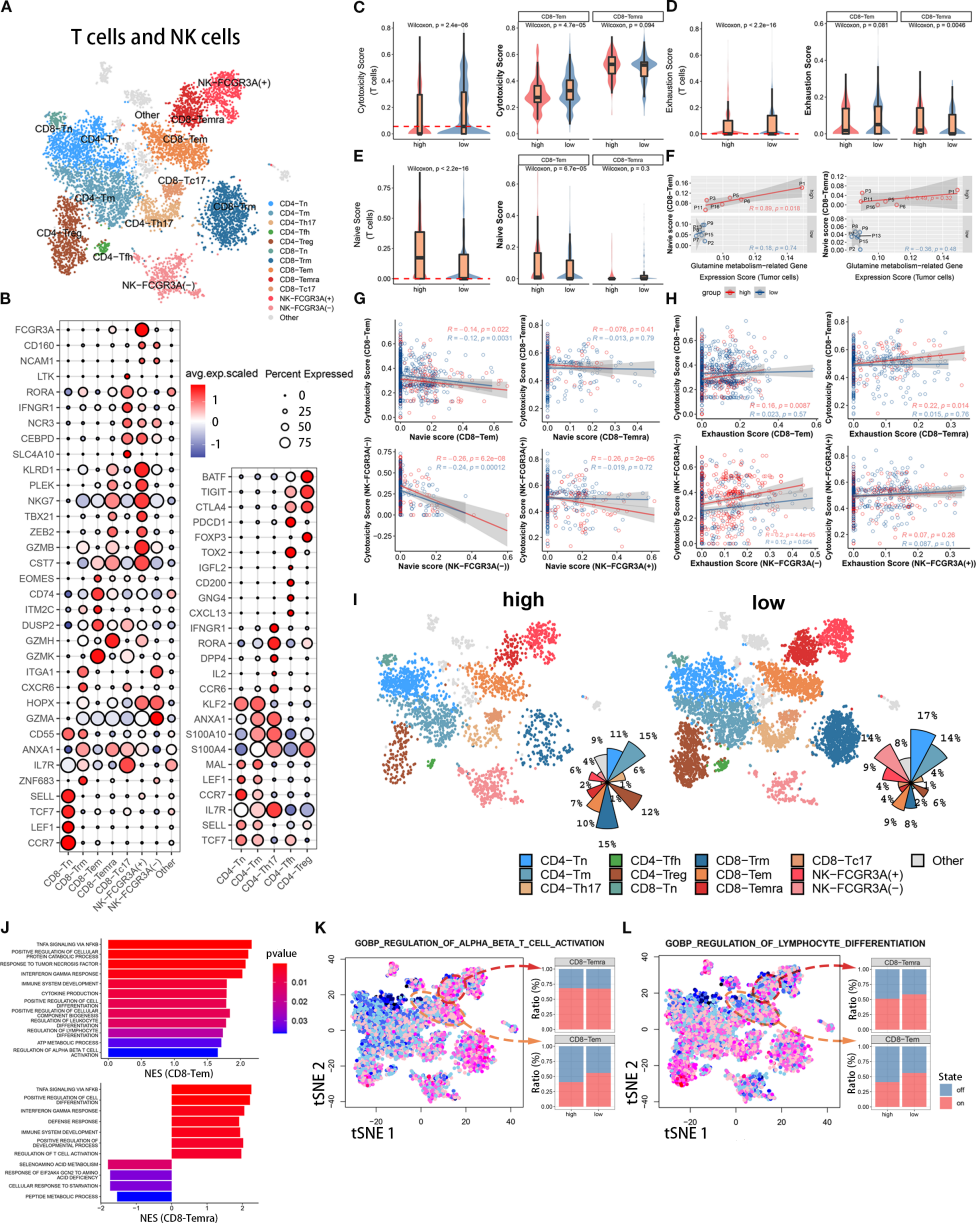
Immune functional differences of CD8+ T cell subsets among different populations. **(A)** The t-SNE plot displays T cell and NK cell subpopulations from the tumor microenvironment of pancreatic cancer patients. Different colors represent different cell types. Subpopulation annotations are shown in the figure. **(B)** The bubble plot shows the expression levels and cell proportions of molecular markers in each cell subpopulation. The color of the dots represents the average expression level of the gene, with red indicating high expression and blue indicating low expression. The size of the dots represents the cell proportion. **(C-E)** These three sets of boxplots show the differences in cell cytotoxicity score, exhaustion score, and naive score of the entire T cell subpopulation and its CD8-Tem and CD8-Temra subpopulations between two groups. **(F)** We show the fitting curve between the tumor cell glutamine metabolism-related gene expression score of the two groups and the naive score of CD8-Tem and CD8-Temra subpopulations. **(G, H)** We separately display the fitting curves between the naive score and exhaustion score and cell cytotoxicity score of four subpopulations (CD8-Tem, CD8-Temra, NK-FCGR3A(-), and NK-FCGR3A(+)) in the high and low metabolism groups. **(I)** Two sets of t-SNE plots and pie charts show the composition ratio of T cell and NK cell subpopulations in two groups. **(J)** The bar graph displays the pathway enrichment differences between CD8-Tem and CD8-Temra subpopulations between the two groups. **(K, L)** The t-SNE plots on the left show the enrichment levels of two pathways in all T cells and NK cells, and the bar graphs on the right show the activation ratio of the two pathways between CD8-Tem and CD8-Temra subpopulations.

To further investigate the effect of tumor cell glutamine metabolism on CD8 effector T cells, we identified 8700 T cells and NK cells into 12 known cell subpopulations and one undefined cell subpopulation based on known molecular markers (**Figures 3A, B**). CD4 T cell subpopulations included CD4Tn (TCF7, SELL, IL7R, CCR7, LEF1, MAL), CD4Trg (FOXP3, PDCD1, CTLA4, TIGIT, BATF), CD4Tm (S100A4, S100A10, ANXA1, IL7R, KLF2), CD4Th17 (CCR6, IL2, DPP4, RORA, IFNGR1), and CD4Tfh (CXCL13, GNG4, CD200, IGFL2, TOX2). CD8 T cell subpopulations included CD8Tem (GZMK, GZMH, DUSP2, ITM2C, CD74, EOMES, CST7), CD8Trm (ZNF683, IL7R, ANXA1, CD55, GZMA, HOPX, CXCR6, ITGA1), CD8Temra (GZMA, GZMH, GZMB, ZEB2, TBX21, NKG7, PLEK, KLRD1), CD8Tc17 (SLC4A10, CEBPD, NCR3, IFNGR1, RORA, LTK), and CD8Tn (CCR7, LEF1, TCF7, SELL). NK cells included NK-FCGR3A(+) cells (NCAM1, CD160, FCGR3A) and NK-FCGR3A(-) cells (NCAM1, CD160). Both CD8Tem and CD8Temra subpopulations belonged to CD8-Tef (**Figures 3A, B**). Some T cells could not be mapped to known molecular markers after grouping, so they were not biologically annotated. The two t-SNE plots and pie charts show the composition ratio of T cell and NK cell subpopulations in the two populations (**Figure 3I**).

#### 3.3.1 Influence of tumor cells glutamine metabolism on CD8 effector T cells cytotoxicity

To investigate the effect of tumor cell glutamine metabolism on CD8 effector T cell cytotoxicity, we calculated a cytotoxicity score for each T cell based on the expression levels of immune cell cytotoxicity-related genes (GZMK, GZMH, GZMB, PRF1, IFNG, EOMES, and NKG7). The cytotoxicity score of T cells in the high glutamine metabolism group of tumor cells was significantly lower than that in the low glutamine metabolism group, and the difference was statistically significant (Wilcoxon test, p = 2.8 × 10−6). Further study of CD8 effector T cells (CD8T-Tem and CD8T-Temra) revealed that the immune cell cytotoxicity score of CD8T-Tem in the high glutamine metabolism group was significantly lower than that in the low glutamine metabolism group, and the difference was statistically significant (Wilcoxon test, p = 4.7 × 10−5). However, there was no statistically significant difference in the immune cell cytotoxicity score of CD8T-Temra between the high and low glutamine metabolism groups (**Figure 3C**). These findings suggest that high tumor cell glutamine metabolism is associated with a decrease in cytotoxicity of the CD8T-Tem subset, while there is no apparent correlation between the cytotoxicity of the CD8T-Temra subset and tumor cell glutamine metabolism.

#### 3.3.2 Influence of tumor cells glutamine metabolism on CD8 effector T cells exhaustion

In order to investigate the impact of tumor cell glutamine metabolism on CD8 effector T cell exhaustion, we calculated an exhaustion score for each T cell based on the expression levels of immune exhaustion-related genes (PDCD1, TIGIT, HAVCR2, LAG3, and CTLA4). In T cells, the immune exhaustion score of the high glutamine metabolism group was significantly lower than that of the low group, with a statistically significant difference (Wilcoxon test, p = 2.2 × 10−16), but this difference was not significant in CD8-Tem (Wilcoxon test, p = 0.081). In CD8-Temra, the immune exhaustion score of the high glutamine metabolism group was actually higher than that of the low group, with a statistically significant difference (Wilcoxon test, p = 0.0046) (**Figure 3D**). This suggests that high glutamine metabolism in tumor cells is associated with exhaustion in the CD8-Temra subset, but not with exhaustion in the CD8-Tem subset.

#### 3.3.3 Influence of tumor cells glutamine metabolism on CD8 effector T cells maturity

In order to investigate the impact of tumor cell glutamine metabolism on the development of CD8 effector T cells, we used immature immune cell-related genes (LEF1, SELL, TCF7, CCR7) to score the immaturity of each T cell. In the high glutamine metabolism group of tumor cells, the immature scores of T cells and CD8-Tem were significantly higher than those in the low score group, with significant statistical differences (Wilcoxon test, p = 2.2 × 10−16, p = 6.7 × 10−5), while there was no statistical difference in immature scores between the two groups of CD8-Temra (**Figure 3E**). We analyzed the fitting curves of tumor cell glutamine metabolism score and immature scores of CD8-Tem and CD8-Temra subsets in the two groups and found that in the high glutamine metabolism group of tumor cells, the immature scores increased with the increase of tumor cell glutamine metabolism score, and the difference in CD8-Tem subset had statistical significance (R = 0.89, p = 0.018) (**Figure 3F**). This indicates that the higher the level of tumor cell glutamine metabolism, the more immature the CD8-Tem subset, and the immaturity level of CD8-Temra subset may not be related to tumor cell glutamine metabolism.

#### 3.3.4 Is there correlation between CD8 effector T cells cytotoxicity and between immaturity scores and exhaustion scores?

The **Figure 3G** shows the fitted curves between the cytotoxicity scores and the immaturity scores of four cell subpopulations (CD8-Tem, CD8-Temra, NK-FCGR3A(-), and NK-FCGR3A(+)) of tumor cells with high and low glutamine scores. The immune cell cytotoxicity of CD8-Tem (high score group: R=-0.14, p=0.022; low score group: R=-0.12, p=0.0031), NK-FCGR3A(-) (high score group: R=-0.26, p=6.2×10−8; low score group: R=-0.24, p=0.00012), and NK-FCGR3A(+) (high score group: R=-0.26, p=2×10−5; low score group: R=-0.019, p=0.72) subpopulations decreased as the immaturity scores increased, especially in the high glutamine score group of tumor cells. However, there was no significant correlation between the cytotoxicity of CD8-Temra subpopulation and the immaturity scores. The **Figure 3H** shows the fitted curves between the cytotoxicity scores of four subpopulations (CD8-Tem, CD8-Temra, NK-FCGR3A(-), and NK-FCGR3A(+)) of tumor cells with high and low glutamine scores and the exhaustion scores. The two t-SNE plots and pie charts show the composition ratio of T cell and NK cell subpopulations in the two populations (**Figure 3I**). The immune cell cytotoxicity of CD8-Temra and NK-FCGR3A(-) subpopulations increased as the exhaustion scores increased, while there was no significant correlation between the cytotoxicity of CD8-Tem and NK-FCGR3A(+) subpopulations and the exhaustion scores. Through the above research, we found that the high metabolism of glutamine in tumor cells may reduce the cytotoxicity of CD8-Tem cells by inhibiting their development and inducing their immaturity. Additionally, the high metabolism of glutamine in tumor cells may promote the exhaustion of CD8-Temra subpopulation. In general, the high metabolism of glutamine in tumor cells is negatively correlated with the anti-tumor activity of CT8 effector T cells.

#### 3.3.5 Influence of tumor cells metabolism on immune cell activation pathways

To further elucidate the mechanism underlying the anti-tumor activity of CD8 effector T cells through the inhibition of tumor cell glutamine metabolism, we used gene set enrichment analysis (GSEA) to quantitatively calculate the pathway enrichment scores of high and low scoring groups in CD8-Tem and CD8-Temra subsets. In the low scoring group, the immune cell activation pathways of CD8-Tem subset were significantly upregulated, such as αβ T cell activation, interferon-gamma pathway, tumor necrosis factor pathway, cytokine production, lymphocyte and leukocyte differentiation, etc. These immune cell activation pathways were also significantly upregulated in the CD8-Temra subset in the low scoring group (**Figure 3J**). In the low scoring group, the T cell activation pathway (CD8-Tem subset) and lymphocyte differentiation pathway (CD8-Tem and CD8-Temra subsets) were significantly higher than those in the high scoring group (**Figures 3K, L**). These studies demonstrate that when tumor cell glutamine metabolism is reduced, the immune activation pathways of CD8-Tem and CD8-Temra subsets are significantly upregulated.

#### 3.3.6 Conclusions

In the microenvironment of pancreatic cancer, high glutamine metabolism in tumor cells has different effects on different CD8Tef subsets. High glutamine metabolism in tumor cells reduces the cytotoxicity and differentiation of CD8-Tem subset, and increases the CD8-Temra exhaustion score. In conclusion, high glutamine metabolism in tumor cells ultimately reduces the anti-tumor activity of CD8-Tef(CD8-Tem and CD8-Temra).

### 3.4 Under the influence of tumor cell glutamine metabolism, CD8 effector T cells have a distinct immune status

**Figure 4:**
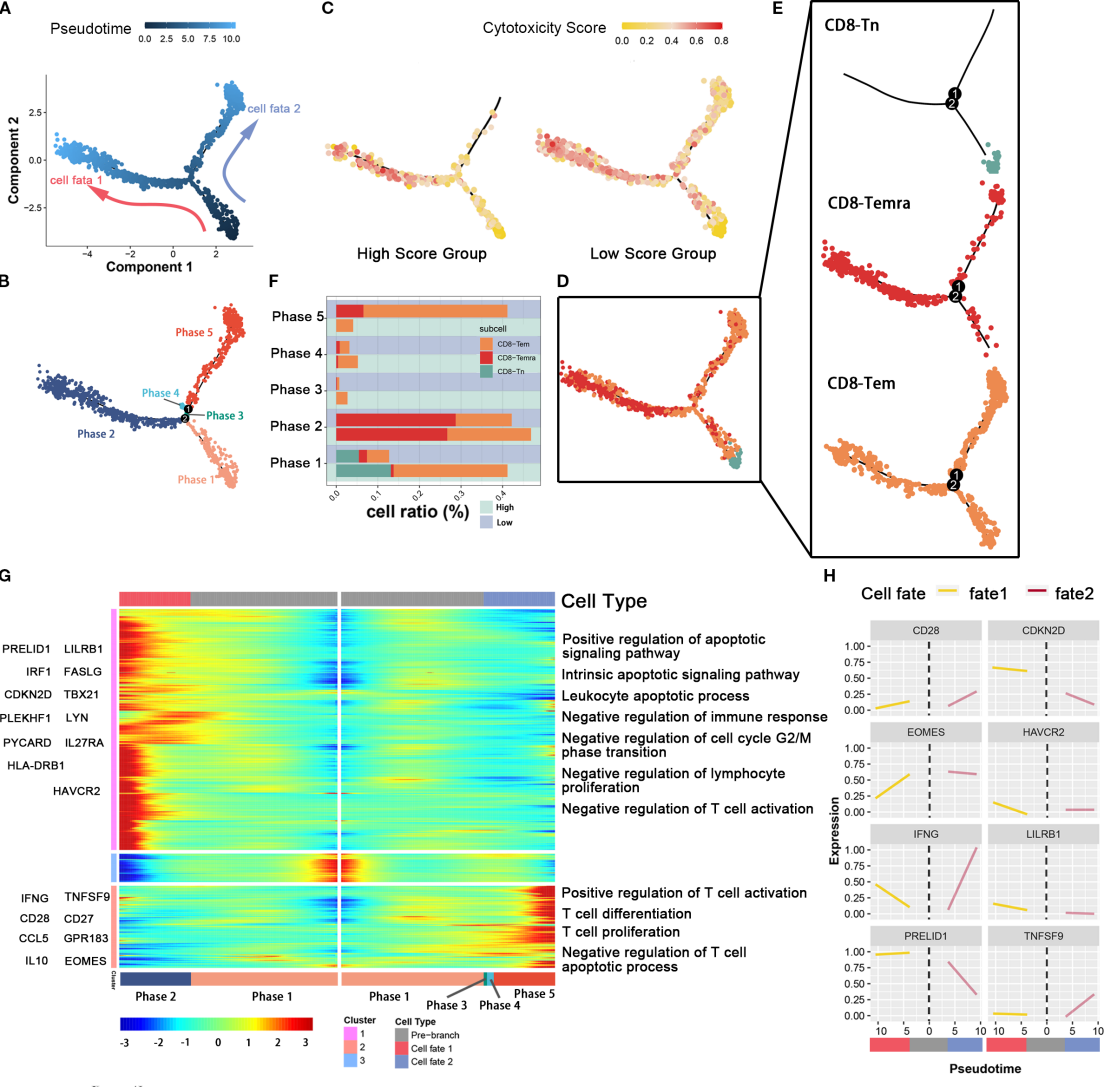
The association between glutamine metabolism of pancreatic cancer cells and the developmental trajectory of CD8+ T cells. **(A)** Pseudotime analysis of CD8-Tem, CD8-Tn, and CD8-Temra subpopulations. Arrows indicate the direction of cell differentiation. **(B)** CD8T cells ordered by pseudotime were divided into five trajectory periods and indicated by different colors. **(C)** Each cell on the pseudotime trajectory was assigned a cytotoxicity score and mapped to the trajectory tree according to color, demonstrating the dynamic changes of cytotoxicity in CD8T cell subpopulations between high metabolic and low metabolic tumor cell groups. **(D, E)** The trajectory tree shows the distribution of three CD8T cell subpopulations along the developmental trajectory. CD8-Tn is indicated by light blue, CD8-Temra by deep red, and CD8-Tem by orange-yellow. **(F)** This histogram shows the cell distribution of CD8-Tn, CD8-Temra, and CD8-Tem subpopulations in five periods among different populations, indicated by different colors for CD8T cell subpopulations. **(G)** The heatmap displays the dynamic changes of gene expression over pseudotime. Representative genes of gene clusters 1 and 2 are listed on the left of the heatmap. Immune regulation-related pathways enriched are labeled on the right of the heatmap. **(H)** The two-dimensional fitting curve shows the dynamic expression of immune-related genes during pseudotime in two developmental pathways.

**Figure 5:**
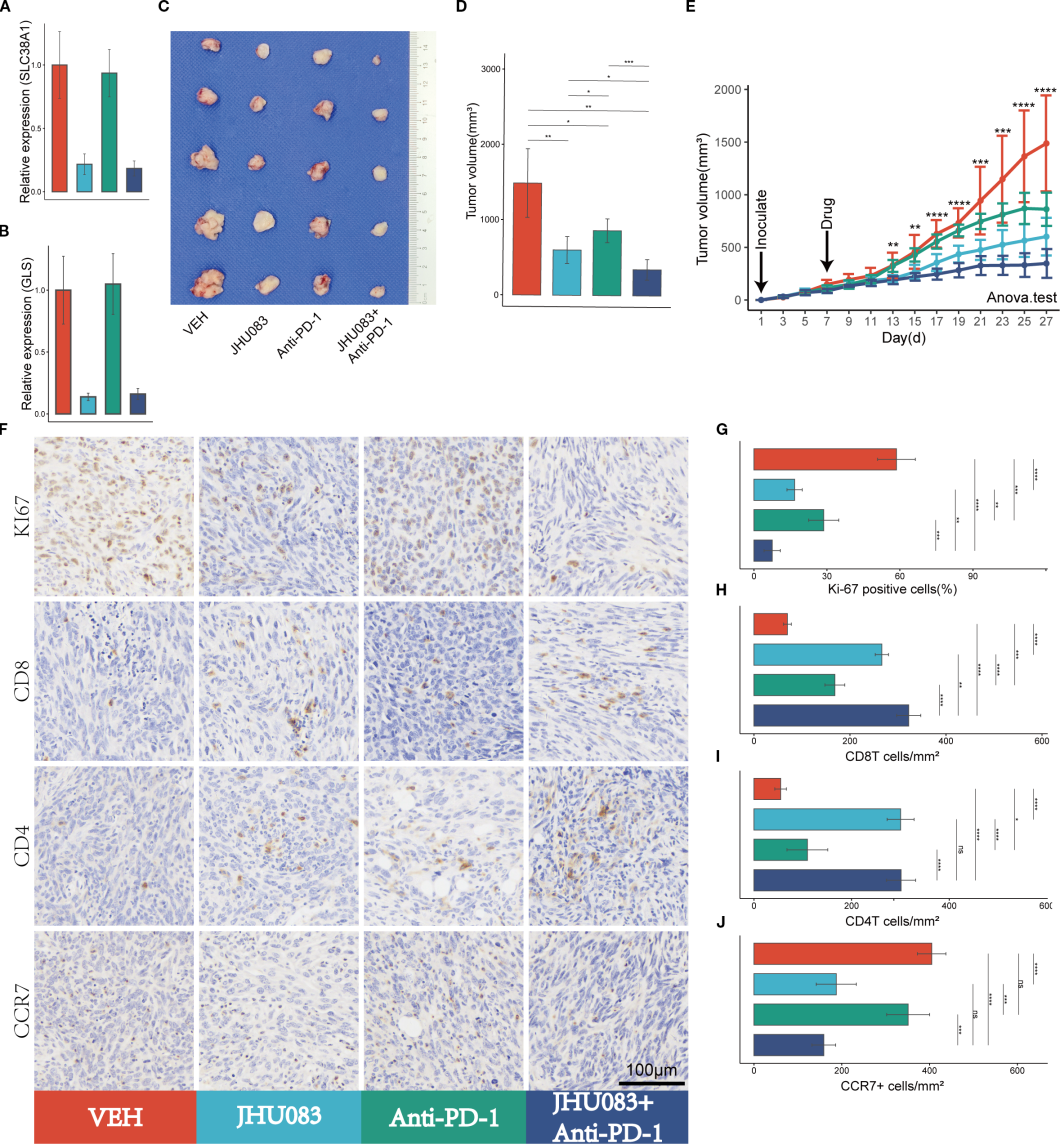
Glutamine metabolism inhibitors (JHU083) exhibit anti-tumor effects and enhance the anti-tumor efficacy of PD-1 inhibitors. Different colors were used to mark the groups: red, blue, green, and purple represent VEH, JHU083, antiPD-1, and JHU083+antiPD-1 groups, respectively. NS indicates no statistical significance; *p < 0.05; **p < 0.01; ***p < 0.001; ****p < 0.0001. **(A, B)** The relative expression levels of SLC38A1 and GLS RNA in tumor tissue after drug treatment. **(C)** Images of mouse tumor specimens collected 26 days after inoculation. **(D)** A bar chart showing the differences in tumor volume among groups of mice collected 26 days after inoculation. **(E)** Tumor growth curves showing the growth rate of tumors in different groups. Panc02 cells were inoculated on day 1 and drug treatment was started on day 7. Tumor volume was measured every 2 days. Significance testing was performed using analysis of variance. **(F)** Immunohistochemical staining of Ki67, CD8, CD3, and CCR7. **(G-J)** Bar charts showing quantitative analysis of immunohistochemical staining for Ki67, CD8, CD4, and CCR7.

In order to study the developmental differences and dynamic changes in genes and pathways of CD8-Tem and CD8-Temra subsets in different levels of tumor cell glutamine metabolism, we used the Monocle package to plot the developmental trajectory of cells (CD8-Tem subset, CD8-Temra subset, and CD8Tn subset) and observed changes in the immune status of CD8 effector T cell subsets. Cells were sequentially arranged on the trajectory tree according to a pseudotime of 0 to 10 (**Figure 4A**), with the early part of the trajectory defined as stage 1. After stage 1, CD8 T cells began to develop in different directions; some cells developed towards stage 2 (fate 1), while others developed towards stages 3, 4, and 5 (fate 2) (**Figure 4B**). Each cell on the pseudotemporal trajectory was scored for cytotoxicity and then mapped to the trajectory tree according to color, showing the dynamic changes in cytotoxicity of CD8T cell subsets between tumor cell high-metabolism and low-metabolism groups (**Figure 4C**). We observed that the cytotoxicity of CD8T cell subsets in the low-glutamine metabolism group of tumor cells was higher than that in the high-glutamine metabolism group at all stages, especially in stages 3, 4, and 5 (fate 2). We also visualized the distribution of CD8-Tem, CD8-Temra, and CD8-Tn subsets on the trajectory tree (**Figures 5D, E**). We calculated the cell proportions of these three T cell subsets at five stages (**Figure 4F**). We observed that the proportion of CD8 T cells in the low-glutamine metabolism group of tumor cells that developed towards fate 2 was significantly higher than that in the high-glutamine metabolism group. There was no significant relationship between the development of CD8 T cells towards fate 1 and tumor cell glutamine metabolism. Pseudotemporal analysis showed that the development of CD8 T cells towards fate 2 mainly upregulated immune activation-related pathways, such as T cell activation pathway, T cell differentiation and proliferation pathway, and T cell apoptosis inhibition pathway, and many pro-immune-related genes, such as CD28, EOMES, INFG, and TNFSF9, were also upregulated. The development of CD8 T cells towards fate 1 mainly upregulated immune inhibition-related pathways, such as immune cell apoptosis activation pathway, inhibition of immune response, inhibition of lymphocyte proliferation, and inhibition of T cell activation, and many genes related to proliferation and immune inhibition, such as PRELID1, LILRB1, CDKN2D, and HAVCR2, were also upregulated (**Figures 4G, H**). In general, the differentiation status and immune function of CD8 T cells exhibit significant heterogeneity between different pancreatic cancer cell glutamine metabolism levels. When tumor cell glutamine metabolism is weaker, CD8 T cells are more likely to acquire stronger anti-tumor activity.

### 3.5 The glutamine metabolism inhibitor JHU083 enhances the anti-tumor effect of immune checkpoint inhibitors (PD-1 inhibitors)

#### 3.5.1 Glutamine metabolism inhibitor JHU083 impact on mRNA expression levels of the glutamine metabolism genes

After treatment with JHU083, the mRNA expression levels of the genes SLC38A1 and GLS decreased significantly, indicating successful inhibition of glutamine metabolism in subcutaneous pancreatic cancer tissue (**Figures 5A, B**).

#### 3.5.2 Glutamine metabolism inhibitor JHU083 impact on tumor volume

To investigate the therapeutic effect of the glutamine metabolism inhibitor JHU083 on pancreatic cancer, we compared the efficacy of four groups of mice treated with different drugs, including the VEH group, JHU083 group, Anti-PD-1 group, and JHU083+Anti-PD-1 group. Compared with the VEH group, both the JHU083 group and the Anti-PD-1 group showed a significant decrease in subcutaneous tumor volume. In addition, the JHU083 group showed a more significant decrease in subcutaneous tumor volume than the Anti-PD-1 group. The combination of JHU083 and PD-1 inhibitor not only significantly inhibited tumor growth but also demonstrated stronger efficacy than using JHU083 or Anti-PD-1 alone (**Figures 5C-E**). These results suggest that JHU083 is effective in treating pancreatic cancer and enhances the anti-tumor effect of immune checkpoint inhibitors (PD-1 inhibitors).

#### 3.5.3 Glutamine metabolism inhibitor JHU083 impact on tumor immune microenvironment

In order to clarify the effect of glutamine metabolism enzyme inhibitor JHU083 on the immune microenvironment of pancreatic cancer, we performed immunohistochemical staining on tumor tissues, including Ki-61, CD8, CD4 and CCR7 (**Figure 5F**). We found that the percentage of Ki-67 positive cells in the JHU083 group, Anti-PD-1 group, and JHU083+Anti-PD-1 group was lower than that in the VEH group, and the difference was statistically significant. Meanwhile, the percentage of Ki-67 positive cells in the JHU083 group was significantly lower than that in the Anti-PD-1 group. The percentage of Ki-67 positive cells in the JHU083+Anti-PD-1 group was significantly lower than that in the JHU083 group and the Anti-PD-1 group, and the difference was statistically significant, indicating that both JHU083 and PD-1 inhibitors can effectively inhibit the proliferation of pancreatic cancer cells. The effect of JHU083 alone was better than that of PD-1 inhibitor alone, and the inhibitory effect of the combination of the two drugs on tumor cell growth was significantly enhanced compared to either drug alone (**Figure 5G**). The CD8T cell density in the JHU083 group, Anti-PD-1 group, and JHU083+Anti-PD-1 group was significantly higher than that in the VEH group, and the difference was statistically significant. The CD8T cell density in the JHU083 group was significantly higher than that in the Anti-PD-1 group, and the CD8T cell density in the JHU083+Anti-PD-1 group was higher than that in either single-drug group (**Figure 5H**). The CD4 T cell density in the JHU083 group, Anti-PD-1 group, and JHU083+Anti-PD-1 group was significantly higher than that in the VEH group, and the difference was statistically significant. The CD8T cell density in the JHU083 group was significantly higher than that in the Anti-PD-1 group, while the CD8T cell density in the JHU083+Anti-PD-1 group was significantly higher than that in the Anti-PD-1 group, with no significant difference from the JHU083 group (**Figure 5I**). This indicates that JHU083 can enhance the immune infiltration of both CD8T and CD4 T cells in the pancreatic cancer microenvironment, while PD-1 inhibitors can only enhance the immune infiltration of CD8 T cells. Compared to JHU083 or PD-1 inhibitor alone, the combination of the two drugs can enhance the infiltration of CD8 T cells in the pancreatic cancer immune microenvironment. There was no statistically significant difference in the CCR7+ cell density between the JHU083 group and the JHU083+Anti-PD-1 group, but it was significantly higher than that in the VEH group and the Anti-PD-1 group, while there was no statistically significant difference in the CCR7+ cell density between the VEH group and the Anti-PD-1 group (**Figure 5J**). This indicates that JHU083 can reduce the proportion of immature T lymphocytes in the tumor immune microenvironment, while PD-1 inhibitors have no such effect. Overall, JHU083 alone has a clear anti-tumor effect on pancreatic cancer and enhances the anti-tumor effect of PD-1 inhibitors.

### 3.6 The glutamine metabolism enzyme inhibitor (JHU083) can inhibit the apoptosis of immune cells in the tumor immune microenvironment and enhance the anti-tumor effect of CD8 T cells

**Figure 6:**
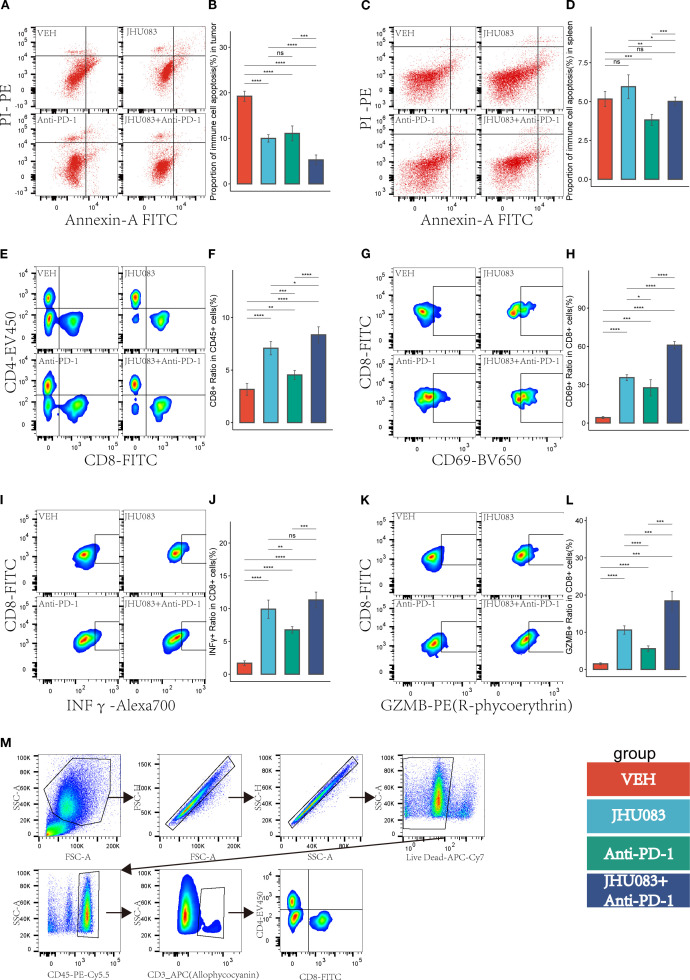
In the pancreatic cancer microenvironment, the inhibition of glutamine metabolism can suppress the apoptosis of immune cells, increase immune cell infiltration, reshape the CD8 T-cell immune phenotype, and enhance the immune therapy response. Different colors were used to mark the groups: red, blue, green, and purple represent VEH, JHU083, antiPD-1, and JHU083+antiPD-1 groups, respectively. NS indicates no statistical significance; *p < 0.05; **p < 0.01; ***p < 0.001; ****p < 0.0001. (**A**, **C**) show the flow cytometry of the tumor microenvironment of subcutaneous tumors in each group of mice and the apoptosis of immune cells in the spleen, respectively. (**B**, **D**) show the statistical analysis of immune cell apoptosis in the tumor microenvironment and spleen, respectively. **(E)** shows the flow cytometry cell sorting diagram, including T cells, CD8+ T cells, and CD4+ T cells. **(F)** shows the difference in the proportion of CD8T cells in CD45 cells in each group. (**G**, **I**, **K**) are flow cytometry cell sorting diagrams, including CD8+CD69+ T cells, CD8+INFγ+ T cells, and CD8+GZMB+ T cells. (**H**, **J**, **L**) show the percentage of the above cells in CD8T cells in a bar graph. **(M)** shows the gating diagram for flow cytometry cell sorting.

In order to investigate the effect of the glutamine metabolism inhibitor JHU083 on CD8 T cells infiltration and immune phenotype in the immune microenvironment, we used flow cytometry to perform immune typing of CD8 T cells in tumor tissue (CD69 as a T cell activation marker, INFγ and GZMB as cell cytotoxicity markers). The proportion of CD8 T cells in CD45 T cells in the JHU083 group, Anti-PD-1 group, and JHU083+Anti-PD-1 group was significantly higher than that in the VEH group. The proportion of CD8 T cells in CD45 T cells in the JHU083 group was significantly higher than that in the Anti-PD-1 group. The proportion of CD8 T cells in CD45 T cells in the JHU083+Anti-PD-1 group was significantly higher than that in the single drug group (**Figures 6E, F**). These results indicate that both JHU083 and Anti-PD-1 can increase the proportion of CD8 T cells in CD45 T cells in the immune microenvironment, and single-use JHU083 is superior to Anti-PD-1, while the combination of the two is better than single drugs. The proportion of CD8+CD69+ T cells in CD8 T cells in the JHU083 group, Anti-PD-1 group, and JHU083+Anti-PD-1 group was significantly higher than that in the VEH group. The proportion of CD8+CD69+ T cells in CD8 T cells in the JHU083 group was significantly higher than that in the Anti-PD-1 group. The proportion of CD8+CD69+T cells in CD8 T cells in the JHU083+Anti-PD-1 group was significantly higher than that in the single drug group (**Figures 6G, H**), indicating that single-use JHU083 and Anti-PD-1 can both stimulate CD8 T cells activation, but single-use JHU083 is superior to Anti-PD-1, and the combination of the two is better than single drugs.

The proportion of CD8+ INFγ+ T cells in CD8 T cells was significantly higher in the JHU083 group, Anti-PD-1 group, and JHU083+Anti-PD-1 group than in the VEH group. The proportion of CD8+ INFγ+ T cells in the JHU083 group was significantly higher than that in the Anti-PD-1 group. The proportion of CD8+ INFγ+ T cells in the JHU083+Anti-PD-1 group was significantly higher than that in the Anti-PD-1 group, but there was no statistically significant difference between the JHU083 group and the JHU083+Anti-PD-1 group. The proportion of CD8+ GZMB+ T cells in CD8 T cells was significantly higher in the JHU083 group, Anti-PD-1 group, and JHU083+Anti-PD-1 group than in the VEH group. The proportion of CD8+ GZMB+ T cells in the JHU083 group was significantly higher than that in the Anti-PD-1 group. The proportion of CD8+ GZMB+ T cells in the JHU083+Anti-PD-1 group was significantly higher than that in the single drug groups. These results indicate that both the JHU083 group and the Anti-PD-1 group can enhance the cytotoxicity of CD8 T cells to a certain extent, but JHU083 alone is superior to Anti-PD-1, and the combination of the two is better than using a single drug. In summary, the glutaminase inhibitor JHU083 can inhibit the apoptosis of immune cells in the tumor immune microenvironment and enhance the anti-tumor effect of CD8 T cells. Furthermore, it can enhance the anti-tumor effect of PD-1 inhibitors.

## 4 Discussion

Pancreatic cancer is known to be glutamine-dependent (41–43), yet how tumor cell glutamine metabolism influences immune cells in the tumor microenvironment is still unclear. In this study, based on the pancreatic cancer dataset in the TCGA database, we found that the tumor glutamine metabolism of patients was negatively correlated with patient prognosis and immune cell cytotoxicity, and positively correlated with immune cell immaturity score. After analyzing the pancreatic cancer single-cell dataset in the GEO database, we found that high glutamine metabolism in tumor cells would inhibit the anti-tumor effect of CD8 T cells. Through *in vivo* experiments in mice, we observed that the glutamine metabolism inhibitor has an anti-tumor effect and can inhibit immune cell apoptosis in the tumor microenvironment, while increasing the cytotoxicity of CD8 T cells and enhancing the anti-tumor efficacy of PD-1 inhibitors.

In recent years, the incidence of pancreatic cancer has been on the rise. It accounts for approximately 2% of all cancers and is associated with 5% of cancer-related deaths (2, 44). The pancreatic cancer microenvironment is considered an immune-suppressive environment (45–51). In the pancreatic cancer microenvironment, most T lymphocytes are CD4 T cells, with CD8 T cells accounting for only a small proportion. The CD4 T cells in the pancreatic cancer microenvironment are mainly Th2 cells, rather than Th1 cells. Th2 cells are associated with tumor immune tolerance, while Th1 cells can increase the tumor-killing effect of CD8 T cells (24, 25). In addition, Treg cells within the CD4 T cell population gradually increase in the development of pancreatic cancer (24, 52). Interestingly, Treg cells play an important role in immune evasion in pancreatic cancer through various immunosuppressive mechanisms (24, 52, 53). These may be the reasons why immune checkpoint inhibitors have not achieved satisfactory therapeutic effects. Therefore, a thorough investigation into the formation mechanism of the immune-suppressive microenvironment in pancreatic cancer is an important approach to improving the efficacy of pancreatic cancer immunotherapy. In this study, through transcriptome sequencing of tissue blocks, we found that the expression score of tumor glutamine metabolism-related genes was negatively correlated with patient prognosis, immune cell toxicity, and immune cell differentiation. Meanwhile, single-cell sequencing data analysis results showed that the anti-tumor activity of CD8T effector cells in the tumor immune microenvironment of patients with high tumor glutamine metabolism was reduced. High tumor glutamine metabolism in tumor cells reduced the cytotoxicity and differentiation degree of CD8-Tem subsets and increased the CD8-Temra exhaustion score. Through GSEA analysis, we observed a negative correlation between tumor cell glutamine metabolism and the activation and differentiation of CD8-Tem and CD8-Temra subsets. Through the above studies, we hypothesize that high tumor glutamine metabolism reshapes the tumor metabolic microenvironment, causing a decrease in the anti-tumor effect of CD8-Tef. Disrupting such abnormal tumor metabolic microenvironments may improve the anti-tumor activity of CD8-Tef, and the efficacy of immune checkpoint inhibitors may also improve.

According to existing research, the tumor microenvironment where CD8 T cells are located is closely related to their developmental trajectory (26). This suggests that the abnormal metabolism of tumor cell glutamine may reshape the metabolic microenvironment of the tumor and alter the developmental trajectory of CD8 T cells. Gene dynamic time analysis and gene set enrichment analysis show that the proportion of CD8 T cells in fate 2 development in the high glutamine score group of tumor cells is significantly lower than that in the low glutamine score group. When CD8 T cells develop into fate 2, they mainly up-regulate immune activation-related pathways. Based on the above data, we speculate that CD8 T cells in the tumor microenvironment with high glutamine metabolism are more likely to lead to weakened anti-tumor activity. When tumor cell glutamine metabolism is inhibited, the developmental trajectory of CD8 T cells returns to normal, and their anti-tumor activity also recovers.

The efficacy of PD-1 inhibitors was found to depend on the infiltration of immune cells in the tumor microenvironment (54, 55). Previous research suggests that blocking the high metabolism of glutamine in tumor cells may increase immune infiltration and promote the differentiation of immune cells, while also potentially enhancing the anti-tumor effect of CD8 T cells. In a subcutaneous pancreatic cancer mouse model, we demonstrated that a glutamine inhibitor can increase the infiltration of CD4 T and CD8 T cells in the tumor microenvironment, promote the differentiation of immune cells, inhibit the rapid proliferation of tumor cells, and enhance the inhibitory effect of PD-1 inhibitors on tumor growth. After treatment with the glutamine inhibitor, the tumor volume significantly decreased, and the growth rate slowed significantly. We found that the anti-tumor effect of using only the glutamine inhibitor was superior to using only PD-1 inhibitor, but the combined use of the two significantly improved the anti-tumor effect. We also observed that the glutamine inhibitor can inhibit the apoptosis of immune cells in the tumor microenvironment, and the combined use of the glutamine inhibitor and PD-1 inhibitor had a stronger effect in inhibiting immune cell apoptosis. Interestingly, the proportion of immune cell apoptosis in the spleen decreased significantly after using only PD-1, but after the combined use of the glutamine inhibitor, the proportion of apoptosis returned to normal levels. Therefore, we speculate that JHU083 not only increases the anti-tumor effect of PD-1 but may also reduce the toxic side effects of PD-1 in normal tissues. Through flow cytometry cell sorting, we found that the glutamine inhibitor can promote the infiltration and activation of CD8 T cells, as well as increase their toxicity, and its effect was significantly enhanced when combined with PD-1 inhibitors. We speculate that the excellent efficacy of JHU083 may be closely related to the increased cytotoxicity of CD8 T cells and the inhibition of tumor cell growth. The effect of the glutamine inhibitor on these two cell subsets has already been confirmed in colon cancer (56). In addition, inhibiting the activity of GLS can reduce the accumulation of intracellular alpha-ketoglutarate and confer a high proliferative and long-lived phenotype to CD8 T cells (56, 57). Even when the glutamine metabolism pathway is completely inhibited, CD8 T cells can still compensate by taking up glucose, increasing the activity of pyruvate carboxylase, and enhancing the activity of the acetyl-CoA metabolism pathway, leading to increased cellular metabolism (58). However, this flexible metabolic compensation mechanism is lacking in tumor cells.

However, our study still has some shortcomings. Although we have demonstrated that inhibiting glutamine metabolism in the tumor microenvironment can increase the infiltration density of CD4 T cells, we have not proven the subtype of CD4 T cells that increased in the tumor microenvironment (TME). Therefore, we cannot determine whether the increased CD4 T cell subtype promotes the enhanced function of CD8 T cells as Th1 cells or promotes immune evasion of pancreatic cancer as Treg cells, or other subtypes. Furthermore, although inhibiting glutamine metabolism in the TME can enhance the cytotoxicity of CD8 T cells, we still do not know the specific mechanism. We have demonstrated that a glutamine inhibitor can enhance the anti-tumor effect of PD-1 inhibitors, but we are not sure whether the enhanced ability of PD-1 to fight tumors is related to the increased cytotoxicity of CD8 T cells. Finally, the animal model we used only includes some pathological and clinical features of human pancreatic cancer, so the sensitizing effect of the glutamine inhibitor on PD-1 inhibitors needs further validation in clinical trials.

## Data Availability

Publicly available datasets were analyzed in this study. This data can be found here: GSE155698, https://www.ncbi.nlm.nih.gov/geo/query/acc.cgi?acc=GSE155698.
