# Supplementary material for: Suppressing glutamine metabolism in the pancreatic cancer microenvironment can enhance the anti-tumor effect of CD8 T cells and promote the efficacy of immunotherapy
Source: Front Immunol. 2025 Oct 2;16:1599252. doi: 10.3389/fimmu.2025.1599252 (PMC12529356; doi:10.3389/fimmu.2025.1599252)
Supplement: Supplementary file 1 [file Table1.docx]

Supplementary Table S1

| Gene | Primer Sequence | |
| --- | --- | --- |
| GLS | Forward | 5'-GCAGTTTGCGTTCCATGTTG-3' |
|  | Reverse | 5'-ATCCAACAATACGCCTCAGTT-3' |
| SLC38A1  GAPDH | Forward | 5'-CGTATGCTCGGGTACCACC-3' |
|  | Reverse  Forward  Reverse | 5'-TGCCTGAGACCCATTCACAC-3'  5'-AATGGGCAGCCGTTAGGAAA-3'  5'-GCCCAATACGACCAAATCAGAG-3' |

Supplementary Table S2

Main Experimental Reagents

| Named Reagents | Manufacturer |
| --- | --- |
| JHU083（GC36368）  Mouse-derived PD1 inhibitor (HRP00262)  CD8（GB13429）  CD4（GB13604-2）  CCR7（GB11502）  KI67（GB111141）  FITC Anti-Mouse CD8a Antibody(E-AB-F1104C)  BV650Anti-Mouse CD69 Antibody(104541)  PE-Cy5.5Anti-Mouse CD45Antibody(35-0451-82)  APC Anti-Mouse CD3Antibody(100236)  EV450 Anti-Mouse CD4Antibody(E-AB-F1097)  ALexa Flour Anti-Mouse INF-γAntibody(557998)  LIVE/DEAD™ Fixable Near IR (780) Viability Kit, for 633 nm excitation(L34992)  eBioscience™ Foxp3/Transcription Factor Staining Buffer Kit (00-5523-00)  eBioscienceTM Annexin V-FITC Apop Kit 100(BMS500fi-300)  PE anti- mouse Granzyme B Recombinant Antibody（372207） | Glpbio  R & D Center of Shanghai Hengrui Medicine Co., Ltd.  Servicebio  Servicebio  Servicebio  Servicebio  Elabscience  Biolegend  eBioscience  Biolegend  Elabscience  BD  eBioscience  eBioscience  Invitrogen  Biolegend |

Supplementary Table S3

| Characteristics | Overall (n=176) | Low Glutamine Metabolism (n=88) | High Glutamine Metabolism (n=88) | p-value |
| --- | --- | --- | --- | --- |
| Age, years | 65.0 (57.0-73.0) | 65.0 (57.0-72.0) | 65.0 (57.0-74.0) | 0.706 |
| Gender, n (%) |  |  |  |  |
| Male | 96 (54.5) | 46(52.3) | 50 (56.8) | 0.648 |
| Female | 80 (45.5) | 42(47.7) | 38 (43.2) |  |
| TNM Stage, n (%) |  |  |  |  |
| I/II | 166(94.3) | 83(94.3) | 83(94.3) | 1.000 |
| III/IV | 10(5.7) | 5 (5.7) | 5 (5.7) |  |
| Tumor size, cm | 3.5(2.95-4.50) | 3.4(2.8.0-4.50) | 3.8(3.00-4.50) | 0.367 |
| Grade, n (%) |  |  |  |  |
| G1-2 | 124(70.5) | 65(73.9) | 59(67.0) | 0.682 |
| G3-4 | 52(29.5) | 23(26.1) | 29(33.0) |  |
